# Dissociation of CAK from Core TFIIH Reveals a Functional Link between XP-G/CS and the TFIIH Disassembly State

**DOI:** 10.1371/journal.pone.0011007

**Published:** 2010-06-08

**Authors:** Hany H. Arab, Gulzar Wani, Alo Ray, Zubair I. Shah, Qianzheng Zhu, Altaf A. Wani

**Affiliations:** 1 Department of Radiology, The Ohio State University, Columbus, Ohio, United States of America; 2 Department of Molecular and Cellular Biochemistry, The Ohio State University, Columbus, Ohio, United States of America; 3 James Cancer Hospital and Solove Research Institute, The Ohio State University, Columbus, Ohio, United States of America; 4 College of Science, King Saud University, Riyadh, Saudi Arabia; University of Minnesota, United States of America

## Abstract

Transcription factor II H (TFIIH) is comprised of core TFIIH and Cdk-activating kinase (CAK) complexes. Here, we investigated the molecular and cellular manifestation of the TFIIH compositional changes by XPG truncation mutations. We showed that both core TFIIH and CAK are rapidly recruited to damage sites in repair-proficient cells. Chromatin immunoprecipitation against TFIIH and CAK components revealed a physical engagement of CAK in nucleotide excision repair (NER). While XPD recruitment to DNA damage was normal, CAK was not recruited in severe XP-G and XP-G/CS cells, indicating that the associations of CAK and XPD to core TFIIH are differentially affected. A CAK inhibition approach showed that CAK activity is not required for assembling pre-incision machinery *in vivo* or for removing genomic photolesions. Instead, CAK is involved in Ser5-phosphorylation and UV-induced degradation of RNA polymerase II. The CAK inhibition impaired transcription from undamaged and UV-damaged reporter, and partially decreased transcription of p53-dependent genes. The overall results demonstrated that a) XP-G/CS mutations affect the disassembly state of TFIIH resulting in the dissociation of CAK, but not XPD from core TFIIH, and b) CAK activity is not essential for global genomic repair but involved in general transcription and damage-induced RNA polymerase II degradation.

## Introduction

The genome of eukaryotic cells is vulnerable to many DNA-damaging agents, which cause devastating cellular consequences. Cells utilize several repair pathways to overcome the deleterious effects of DNA damage and maintain their genome integrity. Nucleotide excision repair (NER) removes a broad variety of double-helix-distorting DNA lesions, including UV-induced cyclobutane pyrimidine dimers (CPD) and 6-4 photoproducts (6-4PP) [1]. NER consists of two sub-pathways: global genomic repair (GGR), which removes DNA damage from the entire genome; and transcription-coupled repair (TCR), which eliminates lesions located on actively transcribed genes [2]. Defects in NER are associated with several rare autosomal recessive genetic disorders, *e.g.*, xeroderma pigmentosum (XP), Cockayne syndrome (CS) and trichothiodystrophy (TTD) [3], emphasizing the important role of NER in maintaining genomic stability.

A generally accepted NER model includes damage recognition, dual incision, and gap-filling DNA synthesis steps [4], [5]. In GGR, the damage-induced DNA distortion is recognized by XPC-hHR23B protein complex [6], [7], and then transcription factor II H (TFIIH) protein complex is recruited via its interaction with XPC to open the DNA helix around the damage site [8]–[10]. In TCR, lesions are detected by RNA polymerase II (RNAP II) in coordination with the recognition of stalled RNAP II by XPG, CSA, CSB and TFIIH [11]–[13]. Other NER factors, such as XPA and RPA, are believed to join the TFIIH-containing repair complex to verify the nature of DNA structure alteration [14]. The endonucleases XPG and XPF-ERCC1 are responsible for the dual incision and the removal of ∼24–32 nt oligonucleotide containing the damage [10]. Subsequent gap-filling DNA synthesis is performed by the concerted action of pol δ or pol ε, and the cofactors PCNA, RF-C and RPA.

Mammalian TFIIH (also referred as holo TFIIH) is organized into core TFIIH, containing the seven subunits XPB, XPD, p62, p52, p44, p34, and p8/TTD-A [15]–[18], coupled to a Cdk-activating kinase (CAK) complex composed of the three subunits Cdk7, cyclin H and MAT1 [19]. TFIIH is a multifunctional protein complex, participating in transcription, NER and cell cycle control [17], [20], [21]. In NER, XPB and XPD helicases of TFIIH are involved in unwinding the DNA duplex around the lesion, providing an open DNA structure for subsequent XPG and XPF-ERCC1 cleavage [10]. During basal transcription, TFIIH functions in harmony with other basal transcription factors, *e.g.*, TFIIB, TFIID, TFIIE and TFIIF. By virtue of its XPB helicase, TFIIH is essential for transcription initiation and promoter escape [22]. In the latter process, Cdk7 of CAK mediates at least partially the phosphorylation of the carboxyl terminal domain (CTD) of the largest Rpb1 subunit of RNAP II [23]. The Cdk7 also mediates the phosphorylation of the activation segment of cyclin-dependent kinases (Cdks), e.g., Cdk2 [24]. Therefore, TFIIH must face the challenge of switching its functional role to cope with its diverse tasks. Such a challenge is obvious in case of TCR, where TFIIH, with help of CSA, CSB and XPG, channels transcription into the process of dual incision in NER [12].

TCR defect is one of the general pathophysiological characteristics of CS and cells derived from CS suffer a global impairment in transcription and exhibit a reduced recovery of RNA synthesis following UV exposure [25]–[27]. CS is caused by mutations in either the CSA or CSB genes [28]. Clinically, CS is characterized by a wide range of symptoms such as severe neurological abnormalities, short stature, lack of subcutaneous fat, hypogonadism, bird-like faces, tooth decay, cataracts and a short lifespan [29], [30]. Some features of CS are shared with TTD, but the latter displays additional defining characteristics. Unlike XP patients, who have extreme sensitivity to sunlight and increased risk of developing sunlight-induced skin cancers, CS patients suffer from skin photosensitivity without cancer predisposition. CS features can exist in combination with XP symptoms (XP/CS) as in XP-B/CS, XP-D/CS, and XP-G/CS cells. Although all XPG mutations result in NER repair deficiency, only C-terminal truncation mutations of XPG lead to CS features in XP-G/CS patients [31]. Additionally, repair-defective XPA and XPF mutations result in XP but not CS symptoms. Thus, NER deficiency cannot explain the CS features of XP-G/CS. The association of XPG with transcription was suggested by the studies of RAD2, a yeast counterpart of XPG [32]. It has been shown that RAD2 plays a role in galactose-induced transcription of GAL7 and GAL10 genes. Yet, the equivalent role of XPG has not been demonstrated in human cells. More recently, it has been reported that XPG forms a stable complex with TFIIH, whereas the truncated XPG proteins in severe XP-G and XP-G/CS patients cannot form an XPG-TFIIH complex [33]. These XPG mutants disturb the interaction of both CAK and XPD with core TFIIH, leading to a defective transactivation of nuclear receptors [33]. In fact, the XPG-TFIIH interaction has been well documented and involves several subunits of TFIIH and at least two regions in XPG [34]–[38]. In spite of the XPG-TFIIH interactions, XPG diffuses freely into the nucleus, and the majority of XPG is not stably associated with TFIIH, but only interacts with other NER components upon recruitment to damaged DNA [39]. More importantly, the XPG-TFIIH interactions and the function of TFIIH are required for recruitment of XPG to DNA damage *in vivo* [36], [37], [39]. Therefore, the biochemical and functional relevance of the disturbed TFIIH to GGR, TCR and transcription, as well as the role CAK in NER *in vivo* remains to be established.

In the present study, we dissected the molecular and cellular manifestation of TFIIH compositional changes in human XPG/CS cells, and explored the role of CAK in removal of UV-induced photolesions, *in vivo* assembly of NER pre-incision complex and transcription. We showed that in repair-proficient cells, both core TFIIH and CAK were rapidly recruited to DNA damage sites and physically engaged in GGR. More importantly, the CAK complex was not recruited to DNA damage in XPG/CS cells, but re-appeared at damage sites in XPG cDNA-corrected XP-G cells. We observed that XPD remains in core TFIIH and is recruited to DNA damage. Using a chemical-genetics based CAK inhibition approach, we were able to further dissect the *in vivo* CAK function in NER and transcription upon UV-induced DNA damage without disturbing the integrity of TFIIH. We found that the kinase activity of CAK complex was not required for *in vivo* assembly of repair machinery or for global genomic repair of UV induced photolesions. Instead, the kinase activity of CAK was involved in the regulation of phosphorylation and UV-induced degradation of RNAP II. Thus, CAK plays a role in general transcription via RNAP II phosphorylation. These results provide insights into a differential functionality of the CAK complex in GGR, TCR and in general transcription *in vivo*, and suggest a role of XPG in retaining association of CAK with core TFIIH. The XPD remaining in the core TFIIH further explains the differences in disassembly state of TFIIH in TTD and CS.

## Results

### Holo TFIIH binds to UV-induced DNA damage and presents in damage sites *in vivo*

**Figure 1:**
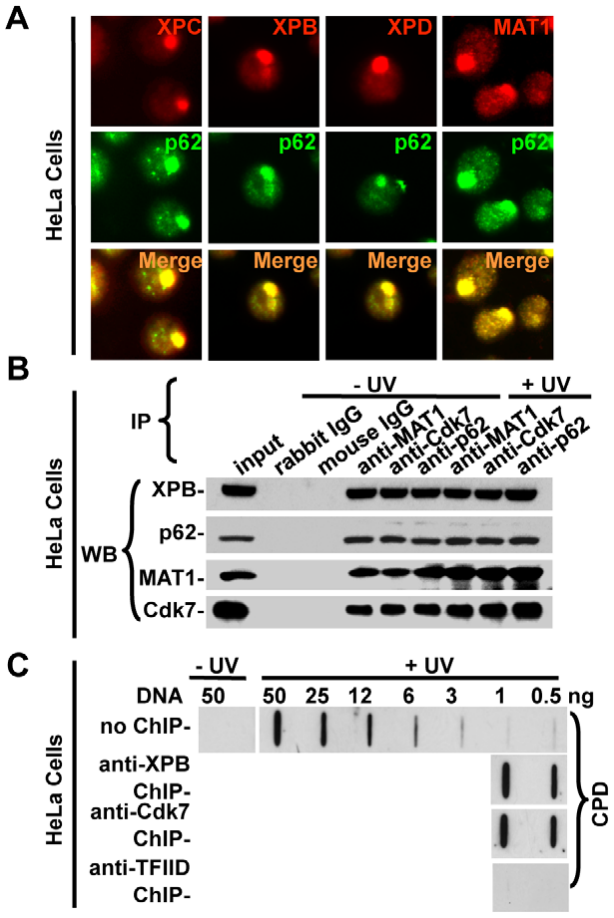
*In vivo* recruitment of holo TFIIH to DNA damage sites in NER-proficient HeLa cells. (**A**) Holo TFIIH is recruited to localized DNA damage sites. HeLa cells were grown on coverslips, irradiated with 100 J/m^2^ UV through a 5 µm isopore polycarbonate filter, cultured for 0.5 h and then fixed with 2% paraformaldehyde. The indicated NER repair factors were visualized by immunofluorescent double labeling using factor-specific antibodies. (**B**) Stable association between core TFIIH and CAK complex in HeLa cells. HeLa cells were either unirradiated or irradiated with 20 J/m^2^ UV and incubated in a fresh medium for 1 h. Whole cell extracts were made and IP was performed using the indicated antibodies. The immunoprecipitates were analyzed by Western blotting for XPB, p62 MAT1 and Cdk7 with specific antibodies. (**C**) Immunoslot-blot analysis of ChIP-recovered DNA. Unirradiated or UV-irradiated (20 J/m^2^) HeLa cells were cultured for 1 h before fixation with 1% formaldehyde. The soluble chromatin was made by sonication and, ChIP was performed with anti-XPB, anti-Cdk7 or anti-TFIID antibodies. The DNA from ChIP was recovered and isolated, and the DNA lesions were detected by immunoslot-blot analysis with anti-CPD antibody. Genomic DNA samples isolated from both unirradiated and UV-irradiated cells were used as controls for estimating the enrichment of UV DNA lesions in ChIP-recovered DNA.

It is well established that holo TFIIH is organized into two subcomplexes, core TFIIH and CAK. Both holo and core TFIIH are functional in dual incision reaction *in vitro* [40], [41]. Yet, the *in vivo* role of CAK in NER remains unproven due to absence of a genetic test of its functions. To approach this question, we first determined which form of TFIIH is engaged in cellular NER by examining the *in vivo* recruitment of core TFIIH and CAK to sub-nuclear spots where DNA damage is locally generated by micropore UV irradiation. The localized DNA damage provokes accumulation/foci formation of NER proteins such as XPC, XPG and TFIIH, which are otherwise uniformly distributed within the nucleus [6], [42]. Immunofluorescence double labeling (Figure 1A) showed that the damage recognition factor XPC, the core TFIIH components XPB and XPD, as well as the CAK component MAT1 were visualized at local nuclear spots, and respectively colocalized with the core TFIIH component p62, indicating the *in vivo* recruitment of holo TFIIH to DNA damage sites. Appearance of CAK together with core TFIIH at damage spots indicated the architectural integrity of TFIIH, which was further confirmed by immunoprecipitation (Figure 1B). Core TFIIH components (XPB and p62) and CAK components (MAT1 and Cdk7) were detected in immunoprecipitates using anti-MAT1, Cdk7 or p62 antibodies in both unirradiated and UV-irradiated cells, and, UV irradiation did not affect the association of CAK and core TFIIH. Consistent with earlier observations [43], these results indicate that core TFIIH and CAK are tightly associated together to form a stable holo TFIIH in repair-proficient HeLa cells.

To investigate whether holo TFIIH specifically binds to UV-damaged DNA *in vivo*, we performed a modified ChIP procedure, which was recently used for detecting the specific binding of yeast Rad26, human CSA and CSB to DNA lesions [13], [44]. The ChIP were carried out, using soluble chromatin obtained from UV-irradiated, formaldehyde-crosslinked HeLa cells and the antibodies against either core TFIIH (anti-XPB) or CAK (anti-Cdk7) subunit. Following ChIP, the bound DNA was recovered, quantified by a sensitive PicoGreen assay, and then examined for CPD lesions. Given that 1 J/m^2^ generates approximately 0.007 photolesions in 1000 bp genomic DNA, ∼5% of chromatin fragments with DNA, ranging from 200 to 600 bp, would contain one photolesion when cells receive 20 J/m^2^ UV irradiation [45]. Thus, ChIP was expected to enrich photolesions about 10–20 times. As expected and as shown in Figure 1C, ChIP against core TFIIH (anti-XPB) and CAK (anti-Cdk7) enriched CPD lesions more than 20 times. Moreover, there was no appreciable difference in photolesion enrichment between anti-XPB ChIP and anti-Cdk7 ChIP. By contrast, ChIP against TFIID, which is not involved in CPD recognition, did not enrich CPD lesions. These results indicated that both anti-XPB and anti-Cdk7 ChIP pull down the TFIIH forms, which specifically bind to DNA damage. In concurrence with CAK and core TFIIH interaction and colocalization, the results further suggested that anti-XPB and anti-Cdk7 ChIP pull down the same holo-TFIIH population. Alternatively, core TFIIH and CAK-containing holo TFIIH are equally capable of binding the damaged DNA. Taken together, these data affirmed the specific binding of TFIIH to DNA damage and revealed the architectural integrity of TFIIH in repair proficient cells.

### *In vivo* CAK recruitment to damage sites is defective in XPG-deficient cells

**Figure 2:**
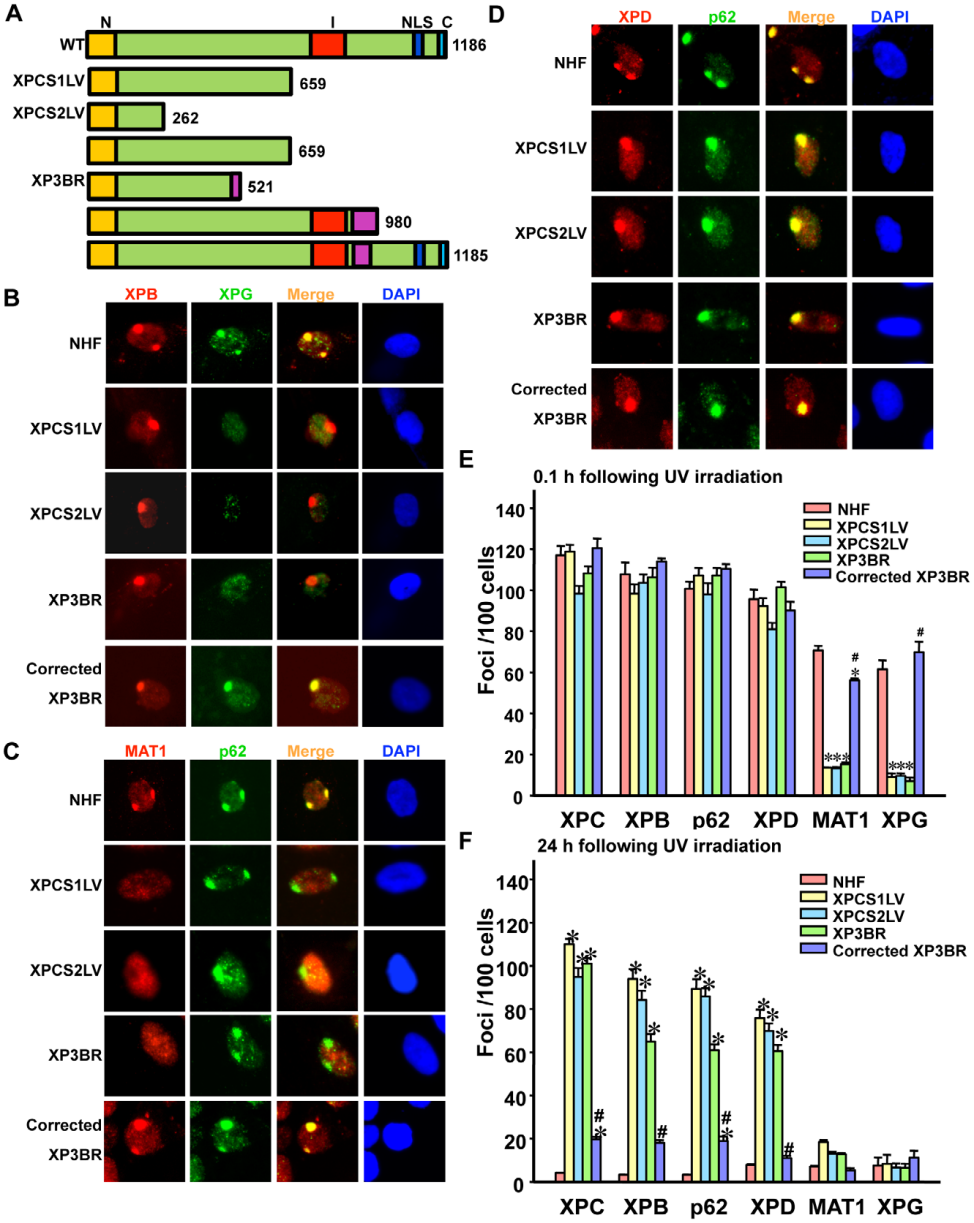
Defective *in vivo* recruitment of CAK to DNA damage sites in severe XP-G and XP-G/CS cells. (**A**) Schematic representation of wild-type and mutant XPG proteins expressed in XPCS1LV, XPCS2LV and XP3BR fibroblasts. N (orange) and I (red) boxes represent the conserved regions essential for the nuclease activity. NLS (blue) and C (cyan) boxes are nuclear localization signal and conserved C-terminal regions, respectively. The (violet) boxes indicate non-XPG residues in XPB3BR cells. The numbers indicate total amino acids in the XPG protein. (**B–D**) Recruitment of core TFIIH (as depicted by XPB, p62 and XPD), CAK (as depicted by MAT1) and XPG proteins to DNA damage sites. Cells were locally irradiated with 100 J/m^2^ UV, allowed to repair DNA for 0.1 h and then processed as described for Figure 1A. (**E and F**) The repair factor recruitment was examined at 0.1 and 24 h post local UV irradiation, respectively. The number of foci/100 cells was calculated based on counting the foci in at least 100 nuclei from five different microscopic fields. Bars indicate mean ± S.E. Symbols * and # indicate significant difference (p<0.05) from NHF and XP3BR cells, respectively.

It has been reported that XPG forms a stable complex with TFIIH and that the composition of TFIIH is affected by XPG mutations [33]. Tracking the sequential assembly of repair proteins *in vivo* would provide clear insights into TFIIH disassembly state and the related repair events in severe XP-G and XP-G/CS cells. We, therefore, assessed the recruitment of core TFIIH as well as CAK subunit to locally damaged DNA to answer whether the recruitment of CAK to DNA damage requires architectural integrity of TFIIH, or if CAK can be sequentially recruited to DNA damage. As revealed by the immunofluorescent staining of XPB and XPG proteins, the C-terminal truncation mutations in XPG protein [46], [47] (Figure 2A) did not affect the early (0.1 h post-UV) XPB recruitment. As expected, XPG recruitment to DNA damage sites was not detected in severe XP-G (XP3BR) and XP-G/CS (XPCS1LV and XPCS2LV) cells (Figure 2B), since the epitope for 8H7 antibody is missing in truncated XPG protein in these cells. Besides, the XPG recruitment to DNA damage sites is expected to be hindered by C-terminal truncation [37]. More importantly, CAK recruitment to DNA damage sites, as depicted by MAT1, was defective in all three XPG-deficient cells, but not in repair-proficient NHF (Figure 2C). On the other hand, early (0.1 h post-UV) recruitment of the core TFIIH subunit p62 was observed in XPG-deficient cells as well as NHF. Consistent with the proposal that XPG mutation affects TFIIH composition [33], these results indicate that CAK is not recruited to DNA damage in XP-G/CS cells. The results further suggest that CAK itself cannot be sequentially recruited to damage-bound TFIIH, but rather functions as part of holo TFIIH in cellular NER.

It has been demonstrated that XPD bridges CAK and core TFIIH by interacting with MAT1 and p44 of core TFIIH [43], [48] and that the XPD co-exists with CAK in purified protein complexes [49]. A recent study reported the interaction between XPD and core TFIIH is disturbed in severe XP-G and XP-G/CS cells [33]. We, therefore, examined the *in vivo* recruitment of XPD to DNA damage sites to clarify whether XPD remains anchored in the core TFIIH in XPG-deficient cells (Figure 2D). Contrary to previous observations, the co-localization of XPD and p62 at DNA damage sites in NHF as well as XPG-deficient cells revealed that XPD was still attached to core TFIIH in these XPG mutant cells.

The XPB, p62, XPD (core TFIIH), MAT1 (CAK) and XPG recruitment was also examined in corrected XP3BR cells, in which XPG function is restored by stable transfection of XPG cDNA. As expected, XPG and more interestingly MAT1 together with XPB, p62 and XPD were efficiently recruited to damage sites (Figure 2B, C and D). Thus, restoration of XPG function also brought back the normal recruitment of MAT1 to DNA damage sites.

We extensively examined the repair factor recruitment in NHF and XPG-deficient cells at different time points post UV irradiation (Figure 2E and F). In NHF and corrected XP3BR, similar patterns of core TFIIH, CAK and XPG recruitment were observed at 0.1 and 0.5 h (data not shown) repair time points, whereas these repair proteins were redistributed and the foci disappeared 24 h after UV irradiation. Conversely, the XP-G deficient cells showed a defective recruitment of MAT1 and XPG to damage site at any time point. In addition, XPG deficiency rendered the repair factors XPC, XPB, p62 and XPD persistent at damage sites 24 h after UV irradiation. It should be noted that the departure of repair proteins indicated the completion of the repair process, whereas the persistence of repair proteins at damage sites suggested the inability of XPG-deficient cells to perform lesion repair. Overall, these results revealed the permanently defective migration of both CAK and XPG to DNA damage sites after UV irradiation in these XPG-deficient cells. The recruitment and persistence of XPD at damage further suggested that CAK and XPD were differentially affected by XPG/CS mutation.

### Dissociation of CAK from core TFIIH proteins disrupts the TFIIH integrity in XP-G/CS cells

**Figure 3:**
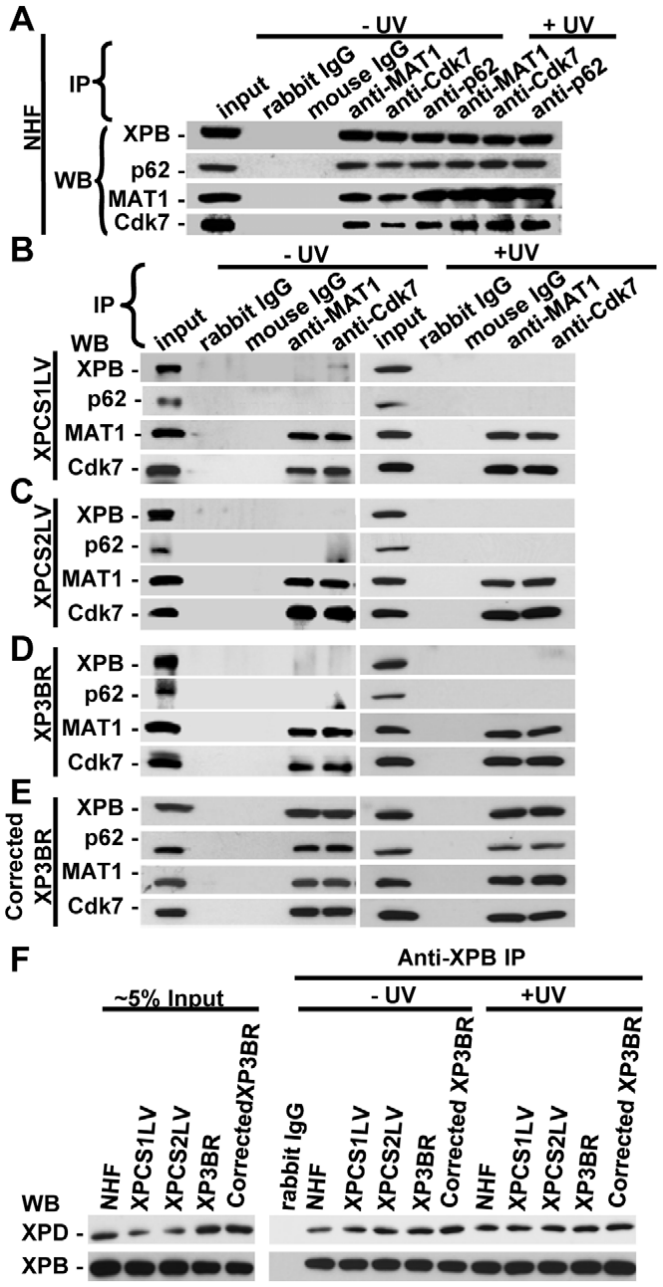
Dissociation of CAK complex from core TFIIH disrupts TFIIH integrity in XPG-deficient cells. (**A**) Tight association between core TFIIH and CAK in NHF. (**B–D**) Dissociation of CAK from core TFIIH occurs in XPCS1LV, XPCS2LV and XP3BR cells with or without UV irradiation. (**E**) Restoration of TFIIH integrity in XPG cDNA-corrected XP3BR cells. (**F**) XPD is present in core TFIIH in XPG-deficient cells regardless of UV irradiation. Whole cell extracts were made from NHF and XPG-deficient cells and IP was performed as described for Figure 1B.

Defective CAK recruitment to damage sites in XP-G/CS cells prompted us to verify the previously reported physical dissociation of CAK from core TFIIH [33]. Our results showed that XPB, p62, MAT1 and CdK7 were all detected in the immunoprecipitates when the IP was performed against either the CAK subunits MAT1 and Cdk7 or the core TFIIH subunit p62 (Figure 3). This indicated that CAK is associated with core TFIIH in NHF as also seen in HeLa cells (Figure 1A and 3A). Again, UV irradiation did not affect the association of CAK and core TFIIH. On the contrary, such an association was disrupted in XPCS1LV, XPCS2LV and XP3BR cells (Figure 3B to D). In these XPG-deficient cells, the XPB and p62 of TFIIH were not detected in anti-MAT1 and anti-Cdk7 immunoprecipitates regardless of UV irradiation. As expected, normal core TFIIH and CAK association was restored in XPG cDNA-corrected XP3BR cells (Figure 3E). Presence of XPD in core TFIIH in XP-G/CS cells was also examined using the IP approach. As shown in Figure 3F, XPD was detected in the anti-XPB immunoprecipitates in NHF, XP-G and cDNA-corrected XP3BR cells. UV irradiation did not alter the association of XPD within core TFIIH. It is proposed, therefore, that XPG mutation causes the dissociation of CAK, but not XPD, from core TFIIH in XP-G/CS cells.

**Figure 4:**
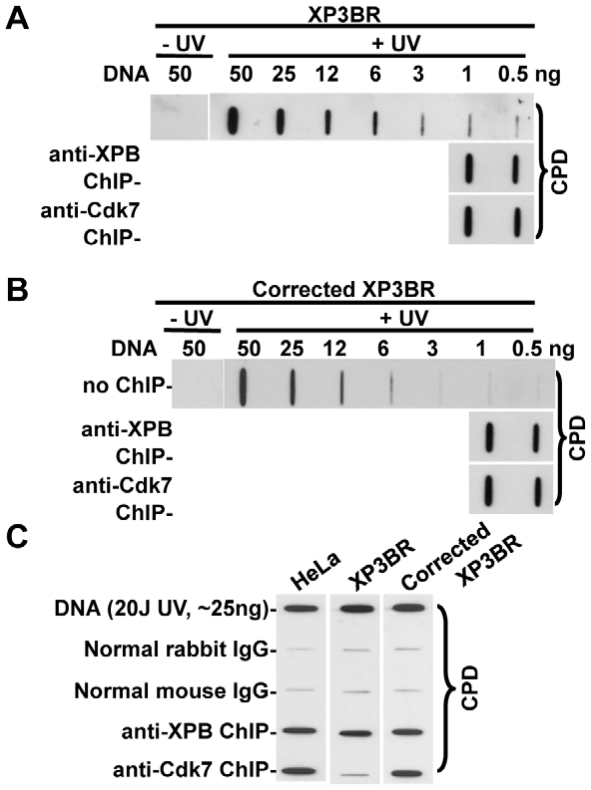
Enrichment of UV-induced photolesions by anti-XPB and anti-Cdk7 ChIP in XP3BR and XPG cDNA-corrected XP3BR cells. The unirradiated or UV-irradiated (20 J/m^2^) fibroblasts were cultured for 1 h to allow DNA repair before fixation. The soluble chromatin preparation, ChIP and DNA isolation were carried out as described in Figure 1C. (**A**) **and** (**B**) Predetermined amount (0.5 and 1.0 ng) of ChIP-recovered DNA was used for Immunoslot-blot analysis of CPD in anti-XPB and anti-Cdk7 ChIP-recovered DNA from XP3BR (**A**) or XPG cDNA-corrected XP3BR (**B**) cells. (**C**) The ChIP-recovered DNA from the same amount of soluble chromatin (500 µg in protein) was used for Immunoslot-blot analysis of CPD in HeLa, XP3BR and XPG cDNA-corrected XP3BR cells. Genomic DNA samples isolated from UV-irradiated cells were used as positive controls.

To investigate whether TFIIH in different forms or disassembly states has different affinity to photolesions, we utilized the ChIP approach, and examined the presence of CPD in ChIP-recovered DNA. When the same amount of ChIP-recovered DNA was analyzed for CPD in XP-G cells, the lesion amounts were comparable in anti-XPB-recovered and anti-Cdk7-recovered DNA in both XP3BR and cDNA-corrected XP3BR cells (Figure 4A and B). These results again indicated that XPB-containing core TFIIH and Cdk7-containing TFIIH were equally capable of binding to CPD, a type of DNA photolesion, which is poorly recognized by NER initiation factor XPC. Surprisingly, CPD was also enriched by anti-Cdk7 ChIP. This result suggests that a small population of holo TFIIH still exists in XP3BR XP-G cells, and again indicated that core and holo TFIIH are equally capable of binding damaged DNA. We addressed this issue further by ChIP experiments in which DNA was recovered from the identical amounts of chromatin (Figure 4C). In both HeLa and cDNA-corrected XP3BR cells, anti-XPB ChIP and anti-Cdk7 ChIP resulted in the capture of the same amounts of CPD. By contrast, only anti-XPB ChIP was able to capture CPD in XP3BR cells, indicating that Cdk7-containing TFIIH population is minimal in these cells. In light of these IP and ChIP data, it can be concluded that the dissociation of CAK from core TFIIH occurred in XPG-deficient cells, severely decreasing the holo TFIIH population, which in turn led to the defective CAK recruitment to damage sites.

### Cdk7 activity of CAK is not required for GGR

**Figure 5:**
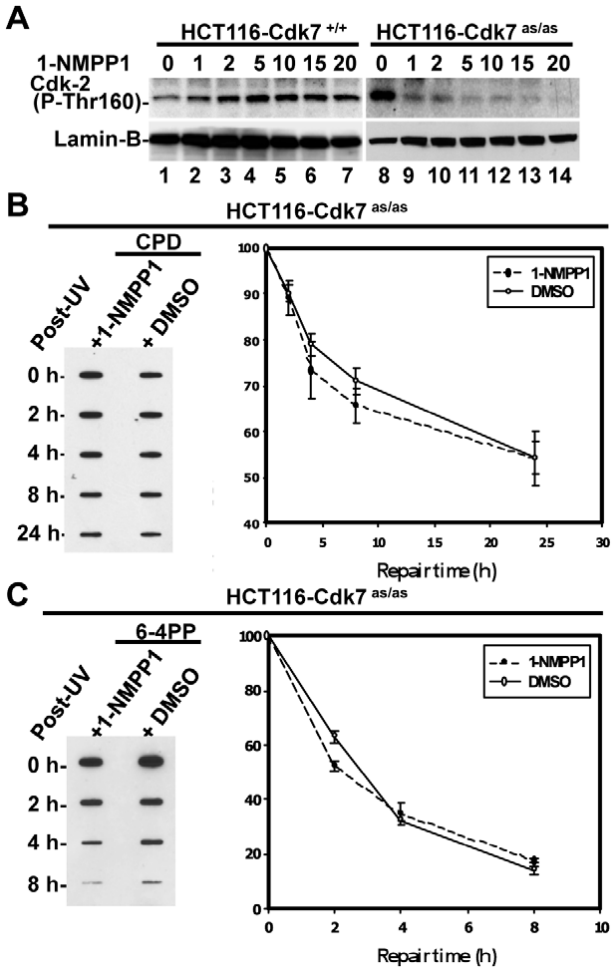
The kinase activity of CAK is dispensable for GGR. (**A**) Dose-dependent inhibition of Cdk7 kinase activity abolishes Cdk2 phosphorylation *in vivo*. Asynchronous HCT116-Cdk7^+/+^ and HCT116-Cdk7^as/as^ cells were incubated 14 h with the indicated concentrations of 1-NMPP1, and the cell lysates were analyzed by Western blotting using anti-phospho-Cdk2 (P-Thr160) antibody. The cellular protein lamin B serves as a loading control. (**B and C**) CAK inhibition does not affect the removal of CPD and 6-4PP from the genome. HCT116-Cdk7^as/as^ cells were starved in serum-free medium overnight and the cells were pretreated with 1-NMPP1 (10 µM) or DMSO (vehicle) for 14 h. The cells were then UV-irradiated with 20 J/m^2^ and allowed to repair DNA in fresh medium with similar composition to the pretreatment for the indicated times. Identical amounts of genomic DNA were subjected to immuoslot-blot analysis of CPD and 6-4PP using the corresponding antibodies. The quantitative data presented in the graph indicate mean ± S.E. of the remaining CPD or 6-4PP from three independent experiments.

Given the defective CAK recruitment to damage sites as a result of disrupted TFIIH integrity in XPG-deficient cells, it is crucial to investigate the *in vivo* functional contribution of CAK to NER. To do so, we used a recently developed chemical genetic testing system where the two wild-type alleles of *Cdk7* gene in HCT116 were replaced with mutant alleles engineered to accommodate bulky, unnatural ATP analogs in their enzymatic active sites in HCT116-Cdk7^as/as^ cells [50]. The mutation of Phe91 to Gly in Cdk7 led to an expansion of the ATP binding pocket, rendering the kinase analog selective and sensitive (as). We first tested the *in vivo* inhibition of Cdk7 in HCT116-Cdk7^as/as^ cells by the ATP analog 1-NMPP1, a potent and selective ATP-competitive mutant kinase inhibitor. Both HCT116-Cdk7^+/+^ and HCT116-Cdk7^as/as^ cells were treated with 1-NMPP1 or the vehicle DMSO for 14 h, and phospho-Cdk2, a biological downstream target of Cdk7, was examined. As shown in Figure 5A, the phospho-Cdk2 level in parent HCT116-Cdk7^+/+^ was not affected by 1-NMPP1 or DMSO treatment. On the contrary, in HCT116-Cdk7^as/as^ cells, a dose-dependent decrease in phospho-Cdk2 occurred upon 1-NMPP1 treatment, with a dramatic reduction in phospho-Cdk2 at the dose of 5 µM and beyond. Next, we assessed the effect of 10 µM 1-NMPP1-mediated Cdk7 inhibition on the repair of UV-induced photolesions. As shown in Figure 5B and C, the removal of both CPD and 6-4PP was not noticeably altered by 1-NMPP1 treatment, indicating a non-essential role of Cdk7 kinase in cellular GGR.

**Figure 6:**
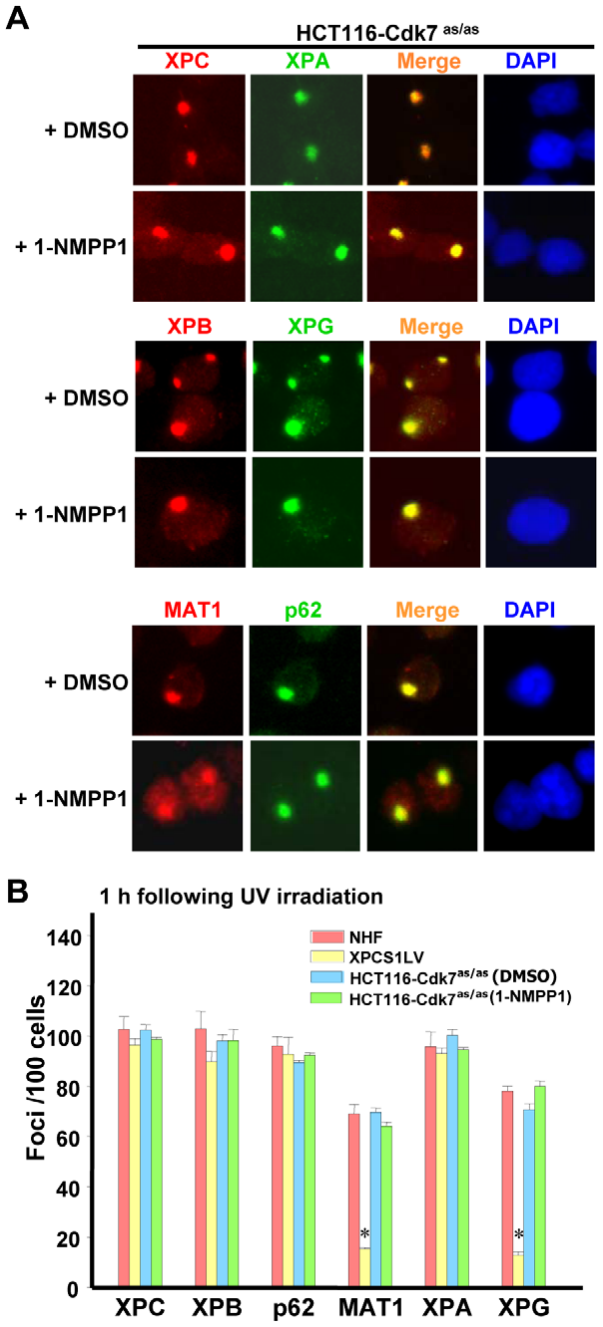
CAK is not essential for the recruitment of XPC, TFIIH, XPA and XPG to DNA damage sites. (**A**) Recruitment of XPC, TFIIH (XPB, p62 and MAT1), XPA and XPG to locally damaged DNA sites is not affected by 1-NMPP1-mediated CAK inhibition. HCT116-Cdk7^as/as^ cells were grown on coverslips and pretreated with 1-NMPP1 or DMSO for 14 h. The cells were then UV irradiated at 100 J/m^2^ through a 5 µm isopore polycarbonate filter, and incubated for another 1 h in fresh medium similar to the pretreatment. The cells were subsequently fixed with 2% paraformaldehyde and processed for immunofluorescence double labeling. (**B**) The quantitative estimation of repair factor recruitment was based on the number of foci/100 nuclei from five different microscopic fields. Bars indicate mean ± S.E. for the indicated antibody staining. Symbol * indicates significant difference (p<0.05) from NHF.

We further explored the impact of Cdk7 inhibition on the recruitment of core TFIIH and CAK, as well as the repair factors XPC, XPA and XPG to local DNA damage sites. The immunofluorescence double labeling showed no changes in the recruitment of XPC, XPA, XPB, XPG, MAT1 and p62 in HCT116- Cdk7^as/as^ upon Cdk7 inhibition (Figure 6A and B), revealing the normal assembly of repair factors following 1-NMPP1 treatment. Taken together, these results indicate that Cdk7 kinase activity does not play a significant role in cellular GGR, while the CAK is physically recruited to DNA damage as part of holo TFIIH.

### CAK plays a role in transcription upon UV- induced DNA damage

**Figure 7:**
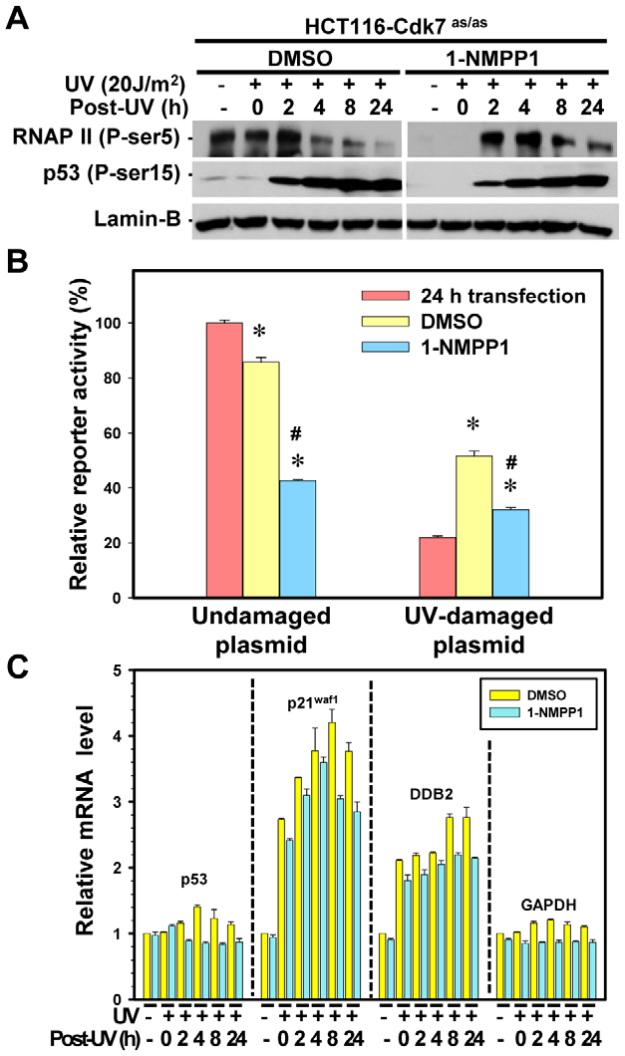
CAK inhibition decreases gene transcription after UV-induced DNA damage. (**A**) Effect of Cdk7 kinase inhibition on the Ser5-phosphorylation of RNAP II. HCT116-Cdk7^as/as^ cells were pretreated with 10 µM 1-NMPP1 or DMSO for 14 h, UV irradiated (20 J/m^2^), and then maintained in fresh medium with the same pretreatment composition for the indicated repair period. The cell lysates were analyzed by Western blotting using anti-phospho-RNAP II and anti-phospho-p53 antibodies. Cellular lamin B serves as a loading control. (**B**) Transcription and transcription recovery measured by Host Cell Reactivation assay with UV-damaged reporter plasmid. HCT116-Cdk7^as/as^ cells were first transfected with undamaged or UV-damaged (1000 J/m^2^) reporter plasmid harboring a CMV-driven luciferase gene for 24 h. After transfection, the cells were maintained in fresh medium containing either 1-NMPP1 or DMSO for another 24 h. The cells were then harvested and the cell lysates were assayed for luciferase activity. The data are expressed as percentage of relative luciferase activity from undamaged reporter 24 h after transfection, and the bars show the calculated mean ± S.E. obtained from at least 4 independent experiments. Symbols * and # indicate significant difference (p<0.05) from 24 h-transfected cells and DMSO-treated transfected cells, respectively. (**C**) Cdk7 inhibition differentially affects transcription and UV-inducible transcription of *p53*, *p21^waf1^*, *DDB2* and *GAPDH* genes. HCT116-Cdk7^as/as^ cells were treated as described in (**A**) and the total RNA was isolated from 1-NMPP1 or DMSO-treated cells. The *p53*, *p21^waf1^*, *DDB2* and *GAPDH* mRNA was detected by real-time RT–PCR assay using gene-specific primers as described in ‘Materials and methods’. The levels of individual mRNA transcripts were expressed relative (fold) to DMSO-treated unirradiated cells as control. Bars represent mean (±SD) of three determinations.

As TFIIH participates in both NER and transcription, we further inspected the impact of CAK inhibition on RNAP II phosphorylation and transcription upon DNA damage to pinpoint the role of CAK in these repair-related events. It was previously reported that siRNA knock-down of Cdk7 impaired UV-dependent transactivation of *p21^waf1^*, *Mdm2* and *ATF3* genes [51]. Yet, knocking down of Cdk7 might affect the architecture of TFIIH, whose integrity is a premise for the functionality in these experiments. Thus, to cautiously dissect CAK function without disturbing the architecture of TFIIH, we again undertook the chemical genetic approach using HCT116-Cdk7^as/as^ cells. Without Cdk7 inhibition, the results showed that the level of Ser5-phosphorylated RNAP II increased slightly 2 h after UV irradiation, and then decreased between 4 and 24 h (Figure 7A). Such a decrease was readily explained by ubiquitin-mediated degradation of the phosphorylated RNAP II [27], [52], [53]. On the other hand, 1-NMPP1 treatment abolished RNAP II Ser5-phosphorylation in the cells without UV irradiation or immediately after UV exposure, indicating that Cdk7 is a major kinase for pre-existing state of RNAP II Ser5-phosphorylation. The phosphorylated RNAP II reappeared 2 h after UV, and the disappearance of the phosphorylated RNAP II upon Cdk7 inhibition was remarkably delayed at 8 and 24 h after UV irradiation. The level of Ser15-phosphorylated p53 progressively increased following UV irradiation indicating the importance of phosphorylation for preventing p53 from ubiquitin-mediated degradation [54]. As expected, Cdk7 inhibition barely reduced the Ser15-phosphorylation of p53 at each time point as compared with DMSO-treated control. Three conclusions can be inferred from these results. First, Cdk7 is the major kinase responsible for pre-existing RNAP II Ser5-phosphorylation without UV irradiation; second, other kinase(s) may be involved in UV induced RNAP II Ser5-phosphorylation when Cdk7 is under inhibition; third, pre-existing Cdk7-mediated RNAP II Ser5-phosphorylation contributes, at least in part, to UV-induced RNAP II degradation.

Next, host cell reactivation (HCR) assay was carried out in HCT116-Cdk7^as/as^ cells to examine the transcription of UV-damaged CMV-driven luciferase reporter. Since UV-induced photolesions strongly block transcription, the recovery of luciferase transcription is considered a result of the removal of photolesions from the transcribing strand of the reporter gene. The results showed that CAK inhibition for 24 h by 1-NMPP1 led to clear reduction (from 85% to 42%) in luciferase activity of undamaged reporter as compared to DMSO mock treatment (Figure 7B). As expected, the UV-induced damage cut the luciferase transcription to ∼22% of undamaged controls. Interestingly, the luciferase transcription recovered and reached ∼52% in DMSO-treated cells, but only ∼32% in 1-NMPP1-treated cells relative to 24 h transfection control. These results indicate that Cdk7 activity of TFIIH is important for transcription of CMV-driven luciferase reporter as well as the UV-damaged reporter.

We further investigated the impact of CAK inhibition on UV-induced transactivation of cellular *p21^waf1^* and *DDB2* genes and their upstream regulator *p53* using real-time RT-PCR. The results showed that p53 mRNA level slightly increased upon UV-induced DNA damage (Figure 7C). Upon CAK inhibition *p53* transcription was unaffected by UV. At the same time, the p21^waf1^ transcription was induced by UV over a period from 2 to 24 h, while DDB2 transcription moderately increased over time. Inhibition of the CAK activity resulted in partial attenuation of mRNA level of both p21^waf1^ and DDB2 over time. For example, the relative p21^waf1^ and DDB2 mRNA were 4.20 and 2.77 at 8 h post-UV. Upon CAK inhibition, these levels decreased to 3.05 and 2.20, respectively. On the other hand, the transcription of glyceraldehyde 3-phosphate dehydrogenase (*GAPDH*) gene was not significantly increased by UV irradiation or decreased by 1-NMPP1 treatment. These results suggest a role of CAK in general transcription and in small part in transcription of UV- inducible genes.

## Discussion

Some of the mutations in XPB, XPD and XPG are known to be responsible for XP as well as XP/CS phenotypes. Yet, the correlation between the molecular alterations of TFIIH and XPG with the disease states is poorly understood. A recent study from Tanaka and Egly laboratories has reported that XPG mutations in XP-G/CS cells affect the assembly states of TFIIH, and that the dissociation of CAK and XPD from core TFIIH leads to a disturbed transactivation of nuclear receptors [33]. In this study, we further demonstrated an *in vivo* physical engagement of CAK in GGR and a functional involvement of CAK in transcription. We showed a defect in CAK, but not XPD recruitment as a distinctive manifestation of the compositional changes of TFIIH in severe XP-G and XP-G/CS cells. We further demonstrated that the Cdk7 kinase of CAK does not play a significant role in GGR, but is involved in the regulation of RNAP II phosphorylation and in the transcription of UV-damaged reporter and UV-induced transcription of p53-dependent genes.

### CAK is physically engaged in cellular GGR as part of holo TFIIH complex but, without an apparent functional commitment to GGR

*In vitro* biochemical activities of TFIIH in NER have been well studied. It has been known that both holo TFIIH and core TFIIH are functionally operative in reconstituted dual incision systems, whereas CAK appeared to negatively regulate the *in vitro* dual incision [5]. Yet, the knowledge of how holo TFIIH, especially CAK complex, participates in NER and/or transcription processes *in vivo* has been lacking. In order to dissect the *in vivo* role of CAK in NER, we first utilized an immunofluorescence approach to directly visualize the recruitment of core TFIIH and CAK to locally damaged DNA. We found that the CAK component MAT1 colocalized with core TFIIH components at DNA damage sites in repair-proficient HeLa (Figure 1A) and NHF (Figure 2B–D) cells. The IP approach revealed that the TFIIH architectural integrity was behind the appearance of holo TFIIH at damage sites. Furthermore, the ChIP against core TFIIH (anti-XPB) and against CAK (anti-Cdk7) equally enriched CPD lesions more than 20 times. It is noteworthy that our immunofluorescence experiments did not rely on isolating TFIIH from its cellular environment at somewhat “physiological conditions”, but directly examined TFIIH by “snapshots” within the cells. Thus, these experiments more likely reflected the “frozen action” of holo TFIIH *in vivo*. We further dissected the *in vivo* role of CAK in NER, using a chemical genetic approach. In a recent report, the siRNA-induced knocking down of Cdk7 caused a marked reduction of both Cdk7 and Cyclin H without affecting the repair activity towards 6-4PP [51]. Compared to knock down experiments, the CAK inhibition approach allowed us to single out the functional impact of Cdk7 without disturbance of TFIIH structure. We found that Cdk7 kinase activity was dispensable for GGR of CPD and 6-4PP (Figure 5B and C) and for the repair factor assembly at DNA damage sites (Figure 6). Our study also suggested that Cdk7 did not play a noticeably negative regulatory role on the overall *in vivo* GGR. Therefore, the core TFIIH is sufficient to support *in vivo* GGR without the contribution of CAK.

### Cdk7 activity of CAK is involved in the regulation of RNAP II phosphorylation, degradation and general transcription

We found that Cdk7 inhibition by 1-NMPP1 effectively eliminated RNAP II Ser5-phosphorylation in HCT116-Cdk7^as/as^ cells without UV irradiation or immediately after UV exposure, which indicated that Cdk7 is the major kinase responsible for RNAP II Ser5-phosphorylation without DNA damage. Interestingly, Cdk7 diminution led to higher levels of Ser5-phosphorylated RNAP II between 4 and 24 h post-UV irradiation. Since kinases, such as Cdk9 and Erk1/2, are also involved in RNAP II Ser5 phosphorylation [55], these kinases may be responsible for RNAP II Ser5 phosphorylation when Cdk7 is under inhibition. Nevertheless, the late disappearance of Ser5 phosphorylated RNAP II indicates a delayed RNAP II degradation upon Cdk7 inhibition, suggesting that the preexisting Ser5 phosphorylation by Cdk7 is important for UV-induced RNAP II degradation. Indeed, Yasukawa and co-workers have recently reported that mammalian Elongin A complex mediates the UV-induced ubiquitination and degradation of the largest subunit Rpb1 of RNAP II [56]. In their study, UV irradiation enhanced the interaction of Elongin A with Ser5, but not Ser2 phosphorylated form or the unphosphorylated form of RNAP II. In accordance, our results suggest that Cdk7 mediated Ser5 phosphorylation plays a significant role in UV-induced degradation of Rpb1 subunit of RNAP II. However, the functional relevance of the degradation of Rpb1 subunit of RNAP II to transcription and DNA repair remains inconclusive. Upon UV irradiation, the elongating RNAP II is arrested by photolesions within the transcribed strand [57], [58], where photolesions are repaired by TCR. It has been demonstrated that ubiquitin-mediated degradation removes the arrested RNAP II from DNA [59], [60]. Such RNAP II removal, thereby, could enable other RNAP II molecules to transcribe the gene. Moreover, the removal of the arrested RNAP II could also allow the TCR machinery to access the damage.

Using HCR assay, we found that Cdk7 inhibition somewhat impaired the transcription from CMV-driven luciferase reporter and the transcription from UV-damaged reporter gene. The observation is readily explained by the role of Cdk7 in transcriptional initiation [55]. Inhibition of the CAK activity resulted in a partial attenuation of mRNA level of both p21waf1 and DDB2 (Figure 7C). This is easily understood by an inhibition of the general transcription in cells. Therefore, CAK may not specifically contribute more to the transcription of the damage-inducible genes. Taken together, these results illustrate the contribution of CAK to general transcription and pinpoint the role of CAK in phosphorylating RNAP II at Ser5 for transcription initiation and ubiquitin-mediated degradation.

### CAK, XPD, TFIIH integrity and the disease phenotypes

We observed that XPG C-terminal truncation disturbed the core TFIIH-CAK interaction, leading to defective CAK recruitment to damage sites. Our study also revealed a normal recruitment of XPD in all XPG-deficient cells tested (Figure 2). This phenomenon was not expected from the previous finding that both CAK and XPD were dissociated from core TFIIH in severe XP-G and XP-G/CS patients [33]. Since XPD cannot be recruited to damage sites as a separate unit apart from core TFIIH, it is conceivable that XPD remained attached to core TFIIH in XPG-deficient cells. At present, we do not know why a disturbed interaction between XPG and TFIIH only affected CAK but not XPD recruitment. Perhaps the immunoprecipitation assay used in the previous study only reflected a relative assessment of core TFIIH-XPD association, and the outcome of the experiment may be also dependent on the experimental conditions. In fact, it was reported that even in XPD mutant HD2 cells, the dissociation of XPD and CAK from other TFIIH subunits was only detected when the immunoprecipitation was performed at high stringency [48]. We propose that XPG mutation may diminish the anchoring of the CAK to XPD of core TFIIH, but not the association of XPD with other TFIIH subunits. Thus, the XPD still behaves as part of core TFIIH inside the cellular environment. Therefore, TFIIH-XPG interaction appears to be important for maintaining the anchoring of CAK to core TFIIH.

Recently, a release of CAK from TFIIH was observed during NER, using chromatin immunoprecipitation [51], and the detachment of CAK from core TFIIH is catalyzed by XPA. It seems that dynamic changes in TFIIH composition occur during DNA repair reaction, coinciding with assembly/release of NER factors. Yet, it remains undetermined whether GGR driven release of CAK from core TFIIH is the cause for CAK dissociation in XP-G/CS cells. Since the return of CAK to core TFIIH on chromatin depends on successful repair, requiring XPG, XPF and other NER factors, we surmise that dynamic change in TFIIH composition consistently and swiftly occurs in cells. XPG protein may catalyze the association of CAK to core TFIIH, thereby maintaining holo TFIIH.

How may various assembly and disassembly states of TFIIH be invoked to explain different phenotypes of XP, XP/CS, or TTD diseases? A simplified model has been proposed to provide an understanding of how the alterations in TFIIH correlate with the disease phenotypes [30]. According to the model, defects in specific enzymatic function of XPB, XPD and XPG typically result in XP. The XP/CS features may result from dissociation of CAK from TFIIH, while a more global destabilization of TFIIH involving XPD dissociation would give rise to TTD. Our observation on XPD's remaining in core TFIIH in XP-G/CS cells provided an experimental support to such a model. Thus, dissociation of CAK from TFIIH would be considered as a distinct molecular feature of XP/CS.

In addition to defective CAK recruitment to DNA damage sites in XP-G/CS, our *in vivo* functional assessment of CAK revealed that core TFIIH was sufficient to support the GGR function, while the kinase activity of CAK contributes to RNAP II phosphorylation. In our study, the inhibition of Cdk7 activity of CAK only partially affected the endogenous damage-induced p53 downstream genes, reflecting the contribution of CAK in general transcription. Thus, the CS features of the XP-G/CS cells may be related to a defect in general transcription, but not specifically in the transcription of damage-inducible genes.

In summary, we have described a manifestation of compositional changes of TFIIH, which are affected by XPG C-terminal truncation mutations, vis-à-vis the assembly of pre-incision complex of NER. We have also depicted a functional involvement of CAK in transcription but not GGR. Our work supported a model which provides basic framework to understand how the alterations in TFIIH correlate with the disease phenotype, especially XP/CS. Whether individual XPG, XPB and XPD mutations affect TFIIH integrity and assembly of pre-incision complex, and whether they are responsible for some of the disease phenotypes warrant additional exploration.

## Materials and Methods

### Cell lines and regents

HeLa-DDB2-XPC cells, stably expressing FLAG-HA epitope-tagged DDB2 and V5-His epitope-tagged XPC, were generated from established HeLa cells, selected with G418 and further sub-cloned by single cell dilution as previously described [61]. The normal human fibroblasts (NHF, OSU-2) were established in our laboratory [62]. Human TERT-immortalized XP-G/CS XPCS1LV cells were provided by Dr. Priscilla K. Cooper (Lawrence Berkeley National Laboratory, CA). Primary XP-G/CS XPCS2LV (GM13370) cells were purchased from the Coriell Cell Repository (Camden, NJ). Severe XP-G XP3BR-SV and XPG cDNA-corrected XP3BR-SV cells were obtained from Dr. Karlene Cimprich (Stanford, CA). HCT116-Cdk7^as/as^ cells, established by replacement of the wild-type Cdk7 with a mutant version sensitive to bulky ATP analogues [50], were provided by Dr. Robert Fisher (Memorial Sloan-Kettering Cancer Center, NY). These cells were grown in DMEM or MEM (for primary XP-G/CS cells) supplemented with 10% FCS, antibiotics and with or without 500 µg/ml G418 at 37°C, in a humidified atmosphere of 5% CO_2_.

1-NMPP1 was purchased from Toronto Research Chemical (Toronto, Canada). Rabbit anti-XPC and anti-CPD antibodies were raised in our laboratory as previously described [61], [63]. Monoclonal anti-6-4PP (64 M-2) antibody was purchased from MBL International (Woburn, MA). Monoclonal anti-XPA (12F5) and anti-XPG (8H7) antibodies were from NeoMarkers (Fremont, CA). Monoclonal phospho-RNAP II antibody (Ser5, clone 4H8) was obtained from Affinity BioReagents (Golden, CO). Rabbit phospho-p53 (Ser15) and phospho-Cdk2 (Thr160) antibodies were from Cell Signaling Technology (Danvers, MA). Rabbit anti-XPB (S-19), anti-XPD (H-150), anti-MAT1 (FL-309), monoclonal anti-p62 (G-10), anti-Cdk7 (C-4) and goat polyclonal anti-Lamin B antibodies were from Santa Cruz Biotechnology (Santa Cruz, CA). Fluorescent conjugated Alexa Fluor 488 (goat anti-mouse) and Texas Red (goat anti-rabbit) were obtained from Invitrogen (Carlsbad, CA) and Santa Cruz Biotechnology, respectively.

### UV and micropore UV irradiation

The cells in a monolayer were washed twice with phosphate-buffered saline (PBS). The UV-C was delivered at a dose rate of 0.5 J/m^2^/sec as measured by Model UVX Digital Radiometer. For micropore UV irradiation, the cells grown on glass coverslips were washed with PBS and a 5 µm isopore polycarbonate filter (Millipore, Bedford, MA) was placed on top of the cell monolayer. The coverslips were irradiated and the cells were processed immediately, or maintained in a suitable medium for the desired period and processed thereafter.

### Immunofluorescence

The immunofluorescence double labeling was performed according to the method established in our laboratory [42], [61]. Briefly, the micropore UV irradiated cells were washed twice with cold PBS, permeabilized with 0.5% Triton X-100/PBS for 8 minutes on ice and then fixed with 2% paraformaldehyde in 0.5% Triton X-100 at 4°C for 30 min. After fixation, the coverslips were rinsed twice with cold PBS and blocked with 20% normal goat serum (NGS) in 0.1% Triton X-100/PBS washing buffer at room temperature for 2 h. Primary rabbit anti-XPC, anti-XPB, anti-XPD, anti-MAT1, monoclonal anti-p62, anti-XPG and anti-XPA antibodies (1∶50 to 1∶1000 dilution), as well as Alexa Fluor 488 and Texas Red-conjugated secondary antibodies (1∶200 to 1∶1000 dilution) were all prepared in washing buffer containing 5% NGS and layered on the coverslips for 1 h at room temperature. Following each antibody incubation step, the cells were washed with 0.1% Tween-20 in PBS 4 times for 5 min each. Fluorescence images were captured with a Nikon Fluorescence Microscope E80i (Nikon, Tokyo, Japan) equipped with SPOT analysis software.

### Immunoprecipitation (IP) and chromatin immunoprecipitation (ChIP)

Cells were grown to ∼70% confluence, left unirradiated or UV-irradiated at 20 J/m^2^ and further incubated for 1 h DNA repair in fresh medium. For IP, the cells were washed twice with PBS, then lysed in lysis buffer (50 mM Tris-HCl [pH 7.8], 150 mM NaCl, 1 mM EDTA, 1% Nonidet P-40, 10 mM 2-mercaptoethanol, 0.5 mM PMSF and a complete protease inhibitor cocktail (Roche Diagnostics, , Indianapolis, IN)). For ChIP, cells were washed twice with PBS and cross-linked with 1% formaldehyde in PBS at room temperature for 10 min, followed by addition of glycine to a final concentration of 125 mM and incubated for 10 min. The cell lysates were subjected to sonication in RIPA buffer (50 mM Tris-HCl [pH, 7.5], 150 mM NaCl, 5 mM EDTA, 1% NP-40, 0.5% sodium deoxycholate and 0.1% SDS, 0.5 mM PMSF and a complete protease inhibitor cocktail) on ice to break the DNA into ∼500 bp fragments. The whole cell extracts (for IP) or chromatin solutions (for ChIP) containing ∼2 mg protein were precleared with protein A/G agarose beads (Calbiochem, San Diego, CA) and then incubated with 2 µg specific antibodies in RIPA buffer at 4°C overnight, followed by addition of 25 µl protein A/G agarose beads and incubation for another 2 h. In IP, the immunoprecipitates were collected and washed 4 times with lysis buffer, re-suspended in 40 µl of Laemmli sample buffer and boiled for 10 min, then subjected to Western Blotting. For ChIP, the immunoprecipitates were successively washed with low salt buffer (20 mM Tris-HCl [pH 8.0], 150 mM NaCl, 0.1% SDS, 1% Triton X-100, 2 mM EDTA), high salt buffer (20 mM Tris-HCl [pH 8.0], 500 mM NaCl, 0.1% SDS, 1% Triton X-100, 2 mM EDTA), LiCl washing buffer (10 mM Tris-HCl [pH 8.0], 250 mM LiCl, 1% NP40, 1% deoxycholate, 1 mM EDTA) and twice with TE buffer (20 mM Tris-HCl [pH 7.5], 1 mM EDTA). The DNA-protein complexes were eluted in a final volume of 300 µl with ChIP elution buffer (1% SDS, 0.1 M NaHCO_3_) and the cross-linking was reversed by adding NaCl to a final concentration 0.2 M and incubation at 65°C for 5 h. The DNA in chromatin immunoprecipitates was recovered by proteinase K and RNase A digestion, followed by phenol/chloroform extraction and ethanol precipitation. The DNA was quantitated with the ultra sensitive PicoGreen reagent (Invitrogen, CA) and subjected to Immunoslot-blot analysis.

### Western Blot and immunoslot-blot analysis

Whole cell extracts were prepared by lysing the cells in lysis buffer (2% SDS, 10% glycerol, 10 mM DTT, 62 mM Tris–HCl [pH 6.8]), supplemented with a protease inhibitor cocktail and phosphatase inhibitor PhosSTOP (Roche Diagnostics)). The proteins, quantitated by DC Bio-Rad Protein Assay, were separated by SDS polyacrylamide gel electrophoresis and transferred to PVDF membrane. The immunoblotting was performed with appropriate primary and secondary antibodies and detected using enhanced chemiluminescence. The immunoslot-blot analysis was performed using either genomic DNA isolated from HCT116-Cdk7^as/as^ cells or ChIP-recovered DNA obtained from HeLa cells after the proper treatments. The CPD and 6-4PP photolesions were quantitated by a non-competitive immunoslot-blot assay as described earlier with some modifications [63]. Identical amounts of DNA were loaded on the nitrocellulose membrane and the damage levels were calculated by comparing the band intensities of the samples with the UV-irradiated DNA standard run in parallel with all the blots.

### Cell transfection and reporter assay

Exponentially growing cells (3×10^5^) were plated in 35 mm dishes ∼20 h before transfection. The cells were transfected with 1 µg of undamaged or 1000 J/m^2^ UV-damaged CMV-Tag2 control plasmid per dish, using Invitrogen Lipofectamine 2000 transfection reagent as instructed by the manufacturer. Following 24 h of transfection, the DNA-Lipofectamine mix was removed and the cultures were supplied with fresh medium containing either 1-NMPP1 or the vehicle DMSO for another 24 h. The cells were then harvested in 100 µl of cell lysis reagent (Promega, Madison, WI) and the luciferase assay was performed and the luminescence was measured with TD-20/20 Luminometer (Turner Designs, Sunnyvale, CA).

### Reverse transcription-polymerase chain reaction (RT-PCR)

Following the desired treatments, HCT116-Cdk7^as/as^ cells were washed with cold PBS and total RNA was isolated with RNeasy Mini Kit (Qiagen, CA). DNAase I digestion was performed to avoid any possible DNA contamination and the RNA was further purified with the Qiagen Kit. The RT-PCR was done by using the Invitrogen SuperScript III first strand-synthesis system as instructed by the manufacturer. Briefly, 1 µg total RNA, 2.5 µM Oligo(dT)_20_ and 500 µM dNTP were incubated in a 10 µl reaction volume at 65°C for 5 min followed by chilling on ice for 1 min. RT buffer, 5 mM MgCl_2_, 10 mM DTT, RNaseOUT (40 U/µl) and SuperScript III RT (200 U/µl) were added to a final 20 µl volume for cDNA synthesis. cDNA products (1 µl each) from RT were subjected to real-time PCR in triplicates with the specific primers for *p53*, *p21^waf1^*, *DDB2*, *GAPDH* and *β-actin* using Roche LightCycler 480 (Applied Science, Indianapolis, IN) and Power SYBR green PCR master mix from Applied Biosystems (Foster City, CA). Relative RNA levels were calculated using comparative method based on ΔCt and ΔΔCt values with β-actin as control. The DNA sequences of used primers were as following: *p21^waf1^* 5′-CCTCAAATCGTCCAGCGACCTT-3′ (forward) and 5′-CATTGTGGGAGGAGCTGTGAAA-3′ (reverse), *DDB2* 5′-CCACCTTCATCAAAGGGATTGG-3′ (forward) and 5′-CTCGGATCT CGCTCTTCTGGTC-3′ (reverse), *p53* *5′*-CACTTGTGCCCTGACTTTCAAAC-3′ (forward) and 5′-ACCAACGGGTCCCAGGGGT-3′ (reverse), as well as GAPDH 5′-GAAGGTGAAGG TCGGAGT-3′ (forward) and 5′-GAAGATGGTGATGGGATTTC-3′ (reverse).
